# German Prize for Dentists’ Exhibits

**Published:** 1883-10

**Authors:** 


### GERMAN PRIZE FOR DENTISTS' EXHIBITS.

At the recent Industrial and Fishery Exhibition at Memel, Germany, the first prize for dental exhibits, a silver medal, was awarded to Dr. Ferrari, an American dentist of that city.—*Ibid.*

